# RT-qPCR detection of dsRNA pesticides: Optimization, limitations, and formulation effects

**DOI:** 10.1016/j.isci.2026.116428

**Published:** 2026-06-18

**Authors:** Venetia Koidou, Evangelia Tamvakologou, Minlee Kim, Dimitrios G. Karpouzas, Kalliope Κ. Papadopoulou, Athanasios Dalakouras

**Affiliations:** 1 ELGO-DIMITRA, Institute of Industrial and Forage Crops, Theofrastou 1, 41335 Larissa, Greece; 2 University of Thessaly, Department of Biochemistry and Biotechnology, Laboratory of Plant and Environmental Biotechnology, Biopolis Campus 415 00, Larissa, Greece; 3 Genolution Inc., 63, Magokjungang 8-ro 3-gil, Gangseo-gu, Seoul, Korea

**Keywords:** Analytical chemistry, Environmental monitoring, Soil science

## Abstract

Double-stranded RNA (dsRNA)-based pesticides are emerging as highly specific and environmentally sustainable crop protection agents. However, their environmental fate remains poorly characterized due to the lack of standardized analytical methods. Here, we developed a quantitative reverse-transcription PCR (RT-qPCR) workflow for quantifying naked and chitosan-formulated dsRNA in laboratory preparations and agricultural soils. Using a 297-bp dsRNA targeting *Leptinotarsa decemlineata* actin (dsACTIN), we assessed nuclease treatment, chitosan formulation, and matrix-matched calibration across three soils differing in pH. Nuclease treatment improved assay specificity, achieving a detection limit of 8 × 10^−8^ ng/L, whereas chitosan formulation decreased analytical sensitivity. Rapid adsorption of dsRNA to soil particles underscored the importance of recovering both soluble and particle-bound fractions. Matrix-matched calibration enabled quantification to 10^−3^ ng dsRNA/g soil. Repeated applications revealed rapid dissipation of naked dsRNA but enhanced persistence of chitosan-formulated dsRNA. Overall, this study provides a framework for environmental monitoring and risk assessment of dsRNA pesticides.

## Highlights

• RT-qPCR workflow established for quantifying naked and chitosan-formulated dsRNA
• Applied dsRNA rapidly adsorbs to soil particles following application
• Matrix-matched calibration improves the accuracy of dsRNA quantification
• Chitosan formulation increases dsRNA persistence in soil

## Graphical abstract

**Figure:**
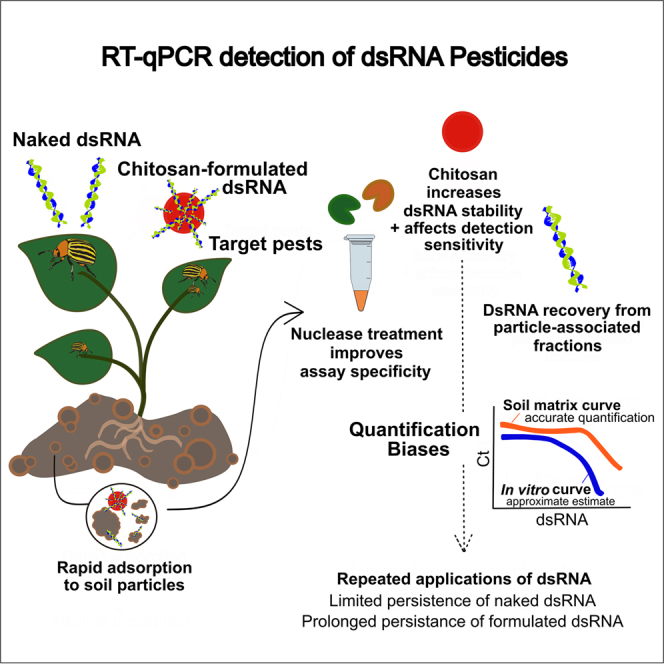

## Introduction

Double-stranded RNA (dsRNA)-based pesticides offer highly specific and environmentally sustainable crop protection by harnessing the mechanism of RNA interference (RNAi). Within the target organism, dsRNA is processed into 20- to 25-nucleotide short interfering RNAs that trigger sequence-specific degradation of essential messenger RNAs, leading to targeted lethality.^1^^,^^2^^,^^3^^,^^4^ This nucleotide-dependent mechanism confers high target specificity and a reduced risk of off-target effects.^5^^,^^6^^,^^7^ Moreover, as inherently labile molecules, dsRNAs typically exhibit limited environmental persistence, thereby conferring a favorable environmental safety profile.^8^^,^^9^^,^^10^ This being said, dsRNA pesticides represent a promising tool for sustainable pest management, enabling effective control with minimal ecological impact.^11^^,^^12^

Quantitative data on dsRNA environmental fate, including persistence, transport, and exposure in soil, water, and plant compartments, are increasingly required for regulatory risk assessment.^9^^,^^13^ Soil is a particularly relevant compartment, acting as both a sink and a potential secondary source of dsRNA, while its microbial and physicochemical characteristics strongly influence degradation and bioavailability.^14^^,^^15^ To enhance stability, uptake, and efficacy, dsRNA is frequently experimentally applied in formulations with inorganic nanoparticles, liposomes, peptide-based carriers, and synthetic polymers.^16^^,^^17^^,^^18^ Experimental dsRNA delivery systems under development often employ nanoparticle-based or cationic carriers, such as chitosan, to enhance stability and uptake. Chitosan, owing to its positive charge, forms efficient electrostatic complexes with negatively charged dsRNA, thereby enhancing stability and cellular uptake, and has thus been widely employed to protect dsRNA from degradation and promote delivery to target organisms.^19^^,^^20^^,^^21^^,^^22^^,^^23^^,^^24^^,^^25^ Yet, while formulations such as chitosan nanoparticles clearly affect RNAi efficiency and environmental fate, their influence on analytical detectability, especially via quantitative reverse-transcription PCR (RT-qPCR), remains poorly characterized.

So far, hybridization-based methods have been widely used for dsRNA quantification, offering specificity but limited versatility, higher costs, and the need for custom probes.^10^^,^^26^^,^^27^ As an alternative, RT-qPCR provides a flexible, cost-effective option with standardized protocols and broad applicability.^15^^,^^28^ Indeed, RT-qPCR has been recently employed for the detection and quantification of exogenously applied dsRNA molecules in water, plants, and agricultural soils.^29^^,^^30^ Nevertheless, accurate dsRNA detection by RT-qPCR is challenged by residual single-stranded RNA (ssRNA) or DNA that may linger in *in vitro* preparations, environmental nucleic acids with partial sequence identity, and inhibitory matrix components such as humic acids, polysaccharides, phenolics, and formulation agents.^31^^,^^32^^,^^33^^,^^34^ Hence, optimized sample preparation, including selective nuclease treatments and purification, seems essential. Calibration strategy is also very critical; standard curves generated in buffer may not reflect amplification in complex matrices, where adsorption, pH, and formulation interactions can alter recovery and amplification efficiency.^35^ Despite this, systematic evaluation of soil matrix-derived standard curves for dsRNA pesticides remains lacking.

In this study, we present a rigorously evaluated and optimized RT-qPCR workflow for the detection of dsRNA in both laboratory (i.e., *in vitro*) and environmental matrices. Specifically, we (1) investigate the effects of nuclease treatment on assay specificity and detection limits, (2) assess the influence of chitosan formulation on RT-qPCR performance, (3) compare *in vitro* and soil matrix-derived standard curves across three distinct agricultural soils for quantification of dsRNA, and (4) examine dsRNA persistence and accumulation under repeated soil applications. To our knowledge, this is the first study to systematically evaluate the combined effects of dsRNA formulation, nuclease treatment, and matrix-dependent calibration across multiple soil types within a unified RT-qPCR analytical framework. Collectively, these analyses establish a standardized framework for RT-qPCR-based accurate quantification of naked and formulated dsRNA pesticides and highlight critical methodological considerations necessary for the assessment of their environmental fate.

## Results

### RT-qPCR detection of dsRNA in *in vitro* samples: Assessing the influence of nucleases and chitosan

**Figure 1:**
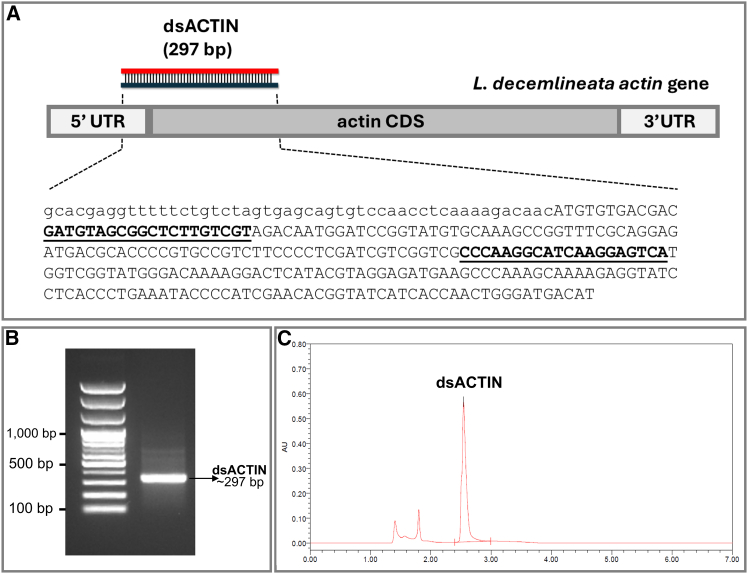
Generation and quality control of dsACTIN (A) The dsACTIN is designed to target a 297-bp fragment of the *Leptinotarsa decemlineata* actin (GenBank: KJ577616.1). The first 49 nucleotides (lowercase) correspond to the 5′ untranslated region (UTR). The binding sites of RT-qPCR primers (bold and underlined) amplify a 121-bp fragment. (B) Agarose gel electrophoresis showing the size and integrity of the *in vitro*-transcribed dsACTIN (right lane); a molecular size marker is shown in the left lane. (C) Reverse-phase HPLC analysis of dsACTIN using a C18 column with UV detection at 260 nm. Separation was performed using an acetonitrile/triethylamine acetate gradient. The purity of the *in vitro*-transcribed dsACTIN was 72.54%.

**Figure 2:**
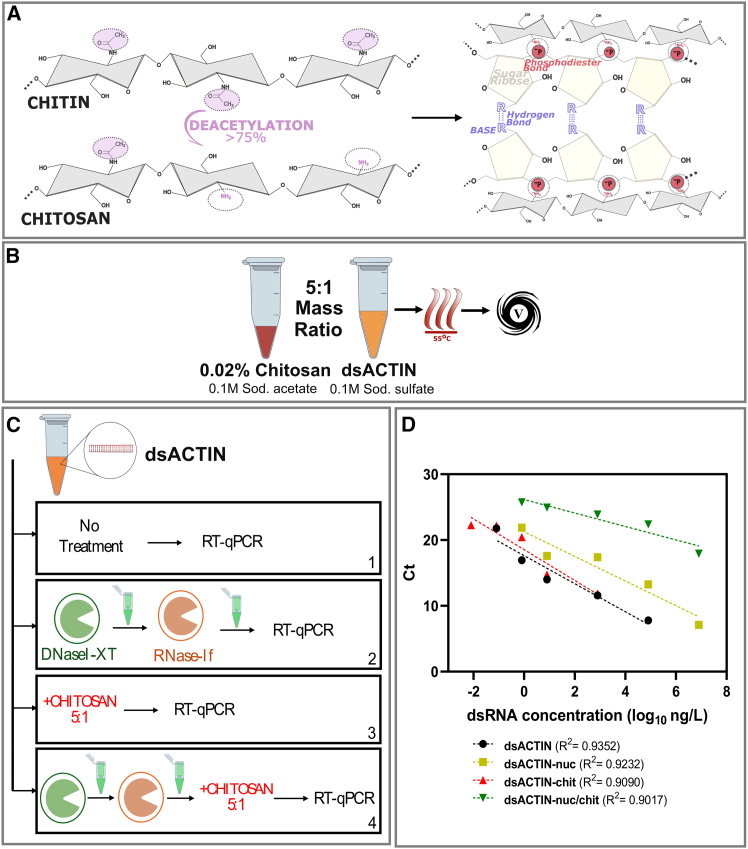
RT-qPCR detection of dsRNA from *in vitro* samples (A) Schematic illustration of electrostatic interactions between chitosan and dsRNA under acidic pH conditions. (B) Schematic overview of the protocol used to generate chitosan-formulated dsACTIN at a 5:1 (w/w) chitosan:dsRNA ratio. (C) Experimental design evaluating the combined effects of nuclease treatment (DNase I-XT and RNase-If) and chitosan formulation on dsRNA detection by RT-qPCR. The following conditions were analyzed: (1) untreated dsACTIN (no nucleases or formulation agent), (2) dsACTIN treated with DNase I-XT and RNase-If (dsACTIN-nuc), (3) chitosan-formulated dsACTIN (dsACTIN-chit), and (4) dsACTIN treated with DNase I-XT and RNase-If and formulated with chitosan (dsACTIN-nuc/chit). (D) *In vitro* standard curves for all treatments (dsACTIN, dsACTIN-nuc, dsACTIN-chit, and dsACTIN-nuc/chit). Ten serial dilutions (8 × 10^6^, 8 × 10^4^, 8 × 10^2^, 8, 8 × 10^−1^, 8 × 10^−2^, 8 × 10^−3^, 8 × 10^−4^, 8 × 10^−6^, and 8 × 10^8^ ng/L) were analyzed for each treatment. Ct values plotted against log_10_-transformed dsRNA concentrations (ng/L), with concentrations expressed on a logarithmic scale, yielded linear regression models used for quantification. Symbols represent measured Ct values, and dashed lines indicate regression fits. Regression parameters, including regression equations, slopes, and corresponding 95% confidence intervals, are provided in Table S4. (A–C) Prepared in Inkscape 1.3.1 (91b66b0, 2023-11-16). The graph in (D) was produced using GraphPad Prism v.10.0.0 for Windows (GraphPad Software, Boston, MA, USA).

The 297-bp dsRNA (dsACTIN) targeting *Leptinotarsa decemlineata actin* (GenBank: KJ577616.1) was synthesized by Genolution Inc. using T7-based *in vitro* transcription and purified via tangential flow filtration.^36^ dsACTIN was stored at −80°C in aliquots as a ready-to-use aqueous solution. The 297-bp dsRNA targeting *Leptinotarsa decemlineata actin* gene (hereafter called “dsACTIN”) (Figure 1A) was produced via *in vitro* transcription (Figures 1B and 1C). To enhance environmental stability, dsRNA delivery systems under development often employ nanoparticle-based or cationic carriers, such as chitosan, to enhance stability and uptake. dsRNA pesticides formulated with chitosan are being experimentally applied to protect dsRNA from degradation and facilitate uptake by target organisms.^19^^,^^20^^,^^25^ At pH < 6.0, the protonated amine groups of chitosan (poly β-1,4-D-glucosamine, >75% deacetylated) interact electrostatically with the anionic phosphate backbone of dsRNA, forming chitosan:dsRNA nanoparticles, whereas at pH > 8.0, dsRNA is largely eluted from chitosan (Figure 2A).^37^ To test the efficiency of chitosan to bind dsACTIN, various chitosan:dsACTIN mass ratios (5:1, 2.5:1, 1.5:1, 1:1, 1:1.5, 1:2.5, and 1:5) were tested in a gel retardation assay; our data suggested that mass ratios ranging from 5:1 up to 1:1 efficiently bound dsACTIN to chitosan nanoparticles (Figure S1). This finding is in agreement with previous reports, where mass ratios of 1.5:1 up to 5:1 have been used for dsRNA formulation.^25^^,^^38^ To evaluate the potential impact of chitosan on RT-qPCR performance, and as a worst-case scenario, we employed a high chitosan:dsRNA mass ratio of 5:1 (Figure 2B), to test whether elevated chitosan concentrations can hinder primer annealing or polymerase activity, potentially reducing amplification efficiency and artificially increasing Ct values. An additional important consideration for accurate dsRNA quantification is that, even after purification, *in vitro*-transcribed dsRNA preparations may still contain residual DNA templates and unannealed ssRNAs; residual impurities inherent to *in vitro* transcription products may contribute to apparent overestimation of dsRNA levels, an effect mitigated by nuclease treatment. In more detail, to address this issue, i.e., evaluate whether nuclease treatment could remove these non-dsRNA molecules and improve RT-qPCR specificity, we used RNase-If, a recombinant fusion protein that selectively degrades ssRNA into mono-, di-, and trinucleotides, and DNase I-XT, an engineered DNase variant that efficiently digests dsDNA, ssDNA, and RNA:DNA hybrids.

Taking the above considerations into account, four *in vitro* sample types were analyzed: (1) naked dsACTIN, (2) nuclease-treated dsACTIN (“dsACTIN-nuc”), (3) chitosan-formulated dsACTIN (mass ratio 5:1) (“dsACTIN-chit”), and (4) chitosan-formulated dsACTIN following nuclease treatment (“dsACTIN-nuc/chit”) (Figure 2C). The effects of chitosan formulation and nuclease treatment on RT-qPCR performance were evaluated in terms of analytical sensitivity, specificity, and quantitative fit. In the absence of both chitosan and nucleases (dsACTIN), the assay exhibited the highest analytical sensitivity, achieving a limit of detection (LOD) of 8 × 10^−8^ ng/L, with moderate fit (R^2^ = 0.9352) (Figure 2D; Table S1). This condition reflects optimal detectability of naked dsRNA but does not account for potential contributions from non-dsRNA templates, which may affect assay specificity. Incorporation of chitosan in the absence of nuclease treatment (dsACTIN-chit) reduced detection sensitivity (LOD = 8 × 10^−4^ ng/L, R^2^ = 0.9090) (Figure 2D; Table S1). This behavior is consistent with partial shielding of dsRNA by chitosan, which may limit template accessibility during reverse transcription and amplification, while non-dsRNA templates remain unremoved. Application of nuclease treatment in the absence of chitosan (dsACTIN-nuc) resulted in an LOD of 8 × 10^−8^ ng/L and an improved fit relative to the chitosan-only condition (R^2^ = 0.9232) (Figure 2D; Table S1). Although the sensitivity was lower compared to untreated naked dsRNA (dsACTIN), nuclease treatment improved the assay specificity by removing non-dsRNA templates, resulting in a more consistent amplification behavior across the dilution series. When chitosan formulation and nuclease treatment were combined (dsACTIN-nuc/chit), sensitivity partially recovered relative to chitosan alone (LOD = 8 × 10^−6^ ng/L), albeit with the lowest fit observed among all conditions (R^2^ = 0.9017) (Figure 2D; Table S1). This outcome suggests that while nuclease treatment improves specificity by eliminating non-target nucleic acids, the combined effects of chitosan complexation and nuclease processing introduce additional variability that constrains quantitative performance. Collectively, these results demonstrate that nuclease treatment primarily enhances RT-qPCR specificity, whereas chitosan formulation exerts a dominant influence on analytical sensitivity. Variations in R^2^ across treatments suggest differences in regression fit and calibration behavior. These differences may be influenced by formulation and enzymatic processing, as well as by matrix-related effects, and highlight the need to carefully consider analytical specificity, sensitivity, and quantification accuracy when optimizing RT-qPCR workflows for formulated dsRNA detection in the various ecotoxicological and environmental fate studies.

### dsRNA detection in soil matrices using *in vitro* standard curves

Because dsRNA persistence is particularly challenged in soil environments, rich in microbial nucleases, we evaluated how nuclease pre-treatment and chitosan formulation influence RT-qPCR detection of dsRNA in soil. To this end, dsACTIN was applied directly to soil to assess recovery efficiency and analytical performance under environmentally relevant conditions. The dsRNA pesticide dose can vary substantially depending on the target pest, crop, formulation, and timing of application. An example application rate reported for a commercial dsRNA product (Calantha), currently the only commercially available dsRNA-based product, targeting *Leptinotarsa decemlineata*, is approximately 10 g per hectare (ha),^39^ corresponding to ∼15 ng dsRNA per g dry soil (ng g^−1^) when assuming a treated soil depth of 0.05 m and a bulk density of 1.5 t m^−3^. With up to four applications per year, the estimated annual input is, therefore, ∼60 ng g^−1^ dry soil, although it needs to be acknowledged that the actual fraction of dsRNA reaching the soil upon application in crops was not directly quantified in our study. To simulate a conservative worst-case exposure scenario, we applied a 40-fold higher dose (2,400 ng g^−1^ dry soil) of naked dsACTIN and chitosan-formulated dsACTIN (dsACTIN-chit) to a sandy loam soil (pH 6.7; organic carbon content 2.9%) collected from a potato field in Kallipeuki, Thessaly. Following soil application, dsRNA distributes between a soluble phase and a fraction associated with soil particles.^15^^,^^40^ To recover the latter, we employed an alkaline extraction buffer (pH 11.0) including orthophosphate ions; at alkaline conditions, dsRNA extraction efficiency is increased, while orthophosphate ions compete with dsRNA for adsorption sites.^10^^,^^15^ In this framework, the aqueous fraction containing dissolved dsRNA is referred to as the “soil solution,” while the fraction recovered after alkaline extraction is termed the “soil extract” (Figure S3A). Both fractions were subjected to nuclease treatment and analyzed by RT-qPCR (Figure S3B) using *in vitro*-derived standard curves (Figure 2D). Because actin is a highly conserved gene and soil matrices may contain nucleic acids with substantial sequence similarity to dsACTIN, we evaluated the specificity of the amplification. To this end, RT-qPCR amplicons were sequenced, confirming the expected target identity and validating the specificity of the method (Figure S2). Yet, quantification of dsRNA in soil using *in vitro* standard curves proved inconsistent, yielding values that either greatly exceeded nominal application doses or were markedly reduced following nuclease treatment (Figures S3C and S3D). Indeed, recent studies have shown that although the use of a single *in vitro* standard curve is operationally convenient, it can significantly compromise quantification accuracy in complex matrices, underscoring that the inclusion of a standard curve in each assay remains the most reliable approach to ensure accuracy and reproducibility^35^ (see below). Despite this shortcoming, our data, importantly, revealed that both dsACTIN and dsACTIN-chit were detected almost exclusively in soil extract fractions, with only negligible amounts recovered from soil solutions (Figures S3C and S3D). These data indicate rapid and extensive adsorption of dsACTIN and dsACTIN-chit to soil particles, consistent with previous reports.^15^ dsRNA adsorption to soil particles is driven primarily by electrostatic interactions between the negatively charged phosphate groups of the RNA backbone and positively charged sites on clay minerals and metal oxides (e.g., Fe^3+^ and Al^3+^) in the soil; multivalent cations such as Ca^2+^ and Mg^2+^ can act as bridges, enhancing binding, while hydrogen bonding between ribose hydroxyls or nucleobases and soil organic matter further stabilizes the complex.^41^ These interactions, combined with physical entrapment within micropores and clay interlayers, reduce dsRNA extractability and partially protect it from enzymatic degradation, influencing its apparent persistence in soil.^41^ This underpins that standard extraction protocols may underestimate actual concentrations if particle-bound dsRNA is not recovered. Additionally, adsorption can partially protect dsRNA from enzymatic degradation, which may lead to apparent persistence in soils. Therefore, optimized extraction methods that recover both soluble and particle-bound dsRNA, combined with matrix-matched calibration curves, are essential for accurate RT-qPCR quantification and for generating reliable soil/environmental exposure data for risk assessment.

### dsRNA detection in soil matrices using matrix standard curves

**Figure 3:**
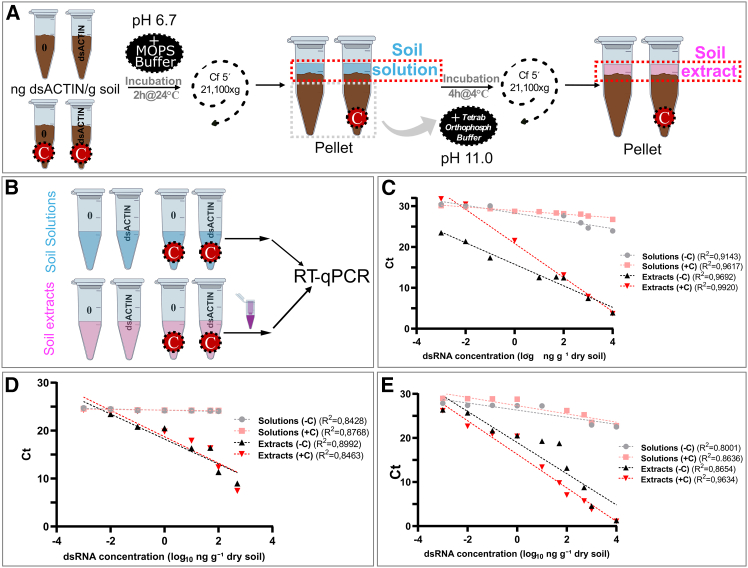
RT-qPCR detection of dsRNA from soil samples (A) Schematic overview of the experimental workflow for dsACTIN application to soil and subsequent recovery from soil matrices. Spiking of graded concentrations of dsACTIN and dsACTIN-chit (10^−3^–10^4^ ng g^−1^ dry soil) was performed to generate matrix standard curves. The dissolved dsRNA was recovered as the soil solution fraction, while adsorbed dsRNA was desorbed using alkaline orthophosphate buffer and recovered as the soil extract fraction. “C” denotes chitosan formulation. (B) Schematic overview of the process followed for dsACTIN and dsACTIN-chit measurement from the two soil fractions. Nuclease treatment step prior to RT-qPCR was omitted. (C–E) Matrix standard curves for naked (−C) dsACTIN and chitosan-formulated (+C) dsACTIN-chit, comparing soil solution and soil extract fractions. A chitosan:dsRNA mass ratio 1.5:1 was used. (C) Acidic topsoil “Ampelies.” (D) Neutral topsoil “Kallipeuki.” (E) Alkaline topsoil “Larissa-University.” Ct values plotted against log_10_-transformed dsRNA concentrations yielded linear regression models used for quantification of experimental samples. Ct values were plotted against log_10_-transformed dsRNA concentrations and fitted using linear regression models within the calibrated concentration range. Symbols represent measured Ct values, and dashed lines indicate regression fits. Regression parameters, including slopes and corresponding 95% confidence intervals, are provided in Table S4. (A and B) Prepared in Inkscape 1.3.1 (91b66b0, 2023-11-16). Graphs were generated using GraphPad Prism.

Based on the above considerations, and to address soil-specific biases in dsACTIN recovery and amplification, we generated matrix-derived standard curves by spiking graded (10^−3^–10^4^ ng_dsRNA_/g_soil_, ng g^−1^) concentrations of dsACTIN and dsACTIN-chit into three agricultural sandy loam soils with contrasting pH (acidic, pH 4.0; neutral, pH 6.7; and alkaline, pH 8.3) and organic carbon content, the two parameters mostly affecting the behavior of organic compounds in the soil matrix. Notably, the chosen chitosan:dsRNA mass ratio was 1.5:1, as this ratio was still sufficient to achieve effective dsRNA formulation (Figure S1), while minimizing potential interactions between excess chitosan and soil-derived inhibitors that could otherwise impair extractability, analytical sensitivity, and assay fit.^42^^,^^43^ Finally, to specifically evaluate formulation effects on soil-based detection, a streamlined protocol omitting nuclease treatment was applied (Figure 3B). As observed previously using the *in vitro* curves (Figures S3C and S3D), dsACTIN was predominantly recovered from extract fractions when the matrix curves were employed (Figures 3C–3E; Table S2), confirming rapid particle association across soil types. The matrix standard curve slopes and amplification efficiencies showed similar trends between dsACTIN and dsACTIN-chit across the tested soils (Figures 3C–3E). While minor variations were observed, these did not indicate a consistent or systematic shift attributable to chitosan formulation. This suggests that chitosan does not markedly interfere with RT-qPCR detection when matrix-specific calibration is employed. Overall, differences in amplification efficiencies observed across soil matrices further highlight the impact of co-extracted inhibitors and matrix-specific effects on RT-qPCR performance. Such deviations from ideal amplification behavior are expected in complex environmental samples and should be interpreted as analytical constraints rather than true differences in target dsRNA quantity.

**Figure 4:**
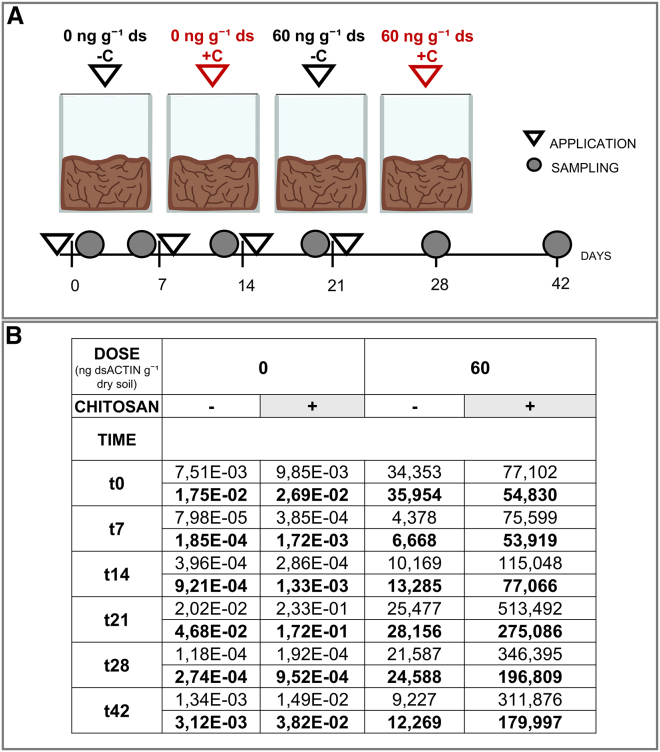
Recovery of naked and chitosan-formulated dsACTIN in soil following repeated applications (A) Schematic depiction of the experimental design for repeated dsACTIN and dsACTIN-chit application to soil. Naked (−C) and chitosan-formulated (+C) dsACTIN and dsACTIN-chit, respectively, were applied sequentially at a concentration of 60 ng g^−1^ dry soil on 0, 7, 14, and 21 days (triangles). Soil samples were collected at six time points: 0, 7, 14, 21, 28, and 42 days (gray-filled circles). A chitosan:dsRNA mass ratio of 1.5:1 was used. (B) Quantification of dsACTIN and dsACTIN-chit recovered from soil extract fractions over time. Results are expressed as ng g^−1^ dry soil. For each treatment, the upper row shows concentrations calculated using *in vitro* standard curves, whereas the lower row (bold values) shows concentrations calculated using matrix-derived standard curves. Quantification of dsACTIN recovered from soil solution fractions was negligible but is presented in Table S3. (A) Prepared in Inkscape 1.3.1 (91b66b0, 2023-11-16).

Repeated applications of a dsRNA pesticide are estimated to be necessary to achieve optimal results.^39^^,^^44^ In an effort to assess the persistence and accumulation of naked and chitosan-formulated dsRNA in soil, we conducted a repeated-application experiment, using dsACTIN and dsACTIN-chit in the soil from Kallipeuki (pH 6.7). dsRNA was applied four times at 0, 7, 14, and 21 days, using 60 ng_dsRNA_/g_soil_ (ng g^−1^) per application, corresponding to the estimated annual field dose of a typical dsRNA pesticide.^39^^,^^44^ Soil samples were collected at 7-day intervals immediately after each application, with additional samples collected at days 28 and 42 without further dsRNA addition (Figure 4A). Samples were fractionated into soil solution and soil extract and analyzed by RT-qPCR across six time points. For the sake of comparison, both *in vitro* (Figure 2D) and Kallipeuki-specific matrix standard curve (Figure 3D) were used for quantification. Consistent with prior observations, dsACTIN and dsACTIN-chit were only minimally detected in soil solution fractions, generally below 10^−3^ ng g^−1^ dry soil when quantified with matrix curves (Table S3). In contrast, soil extract fractions yielded substantially higher recoveries (Figure 4B), confirming that dsRNA rapidly associates with soil particles upon application. Naked dsACTIN showed only modest increases in extractable levels following repeated applications, with overall recovery remaining low, reflecting the expected rapid microbial-based degradation of unformulated dsRNA in soil.^10^^,^^26^ Gradually diminishing dsACTIN was detectable after the final application (day 28), indicating progressive, time-dependent degradation (Figure 4B). In contrast, dsACTIN-chit displayed markedly different behavior. Extractable dsACTIN-chit levels remained relatively stable during the first 2 weeks and increased following the third application (day 21), maintaining elevated plateau levels through days 28 and 42, even in the absence of further additions (Figure 4B). These results indicate that chitosan prolongs dsRNA persistence, likely by promoting protective adsorption to soil particles, thereby increasing extractable recovery over time. While both *in vitro* and matrix-based standard curves revealed dose-dependent accumulation trends, matrix curves attenuated apparent accumulation and revealed a more realistic saturation profile (Figure 4B). Linear regression equations and coefficients of determination (R^2^) were calculated for each standard curve, and regression parameters, including slope and corresponding 95% confidence intervals, are provided in Table S4. The above findings underpin that, although *in vitro* curves may still provide rough estimates, matrix-derived standard curves substantially improve the robustness of dsRNA quantification, in agreement with previous observations.^35^

## Discussion

Accurate characterization of dsRNA pesticide fate is essential for robust environmental risk assessment and informed regulatory decision-making.^5^^,^^9^^,^^14^^,^^15^ Recently, RT-qPCR has been employed by Zarabbian and Sharif to detect and quantify dsRNA pesticides in soil, water, and plant samples, while simultaneously elucidating the complex degradation pathways of both naked and minicell-encapsulated dsRNAs.^29^^,^^30^ Notably, in soil samples, the authors successfully mitigated soil matrix interference, thereby enabling reliable environmental monitoring of dsRNA.^30^ Interestingly, in aquatic systems, they further demonstrated that fungal communities, rather than bacterial populations, exhibit a stronger correlation with dsRNA degradation dynamics.^29^ Similarly, Zhang and co-workers applied RT-qPCR to detect exogenously introduced dsRNA molecules across diverse agricultural soils; a critical determinant of their methodological efficiency was the development of optimized protocols to facilitate the release of dsRNA from adsorbed soil matrices.^15^ In the current study, we systematically evaluated RT-qPCR for the detection of naked and chitosan-formulated dsRNA, focusing on analytical specificity, formulation effects, and matrix-dependent biases. Importantly, our work integrates these methodological parameters within a unified experimental framework, enabling their combined assessment under consistent conditions, which has not been systematically addressed in previous studies. We show that nuclease treatment, although it does not improve detection sensitivity, enhances assay specificity by removing non-dsRNA templates. Importantly, it needs to be noted that RT-qPCR-based detection reflects the presence of amplifiable dsRNA-derived sequences and does not necessarily indicate the persistence of intact or biologically active dsRNA molecules. This parameter may be considered essential in the quantification of dsRNA applied in various ecotoxicological or environmental fate bioassays. Overall, the comparison between nuclease-treated and untreated conditions allows the separation of analytical specificity effects from formulation-driven detection behavior under environmentally relevant conditions. Chitosan formulation prolongs dsRNA persistence in soil but can modulate analytical performance and apparent sensitivity, particularly in combination with nuclease treatment. Importantly, we show that applied dsRNA rapidly associates with soil particles; this finding highlights that neglecting particle-bound fractions results in substantial underestimation of environmental dsRNA levels. However, the bioavailability of particle-associated dsRNA to soil microorganisms remains unclear and requires further investigation, particularly in relation to formulation-dependent protection and degradation dynamics. Our method constitutes a benchmark in the detection and quantification of dsRNA in soil which might require adjustments and further optimization steps depending on the environmental matrix and dsRNA formulation used. Additionally, we demonstrate that *in vitro* standard curves are insufficient for reliable soil quantification and can lead to over- or underestimation of dsRNA concentrations. Consistent with recent findings by Casado-Martín et al.,^35^ matrix-derived standard curves provide more accurate exposure estimates and reveal cumulative patterns under repeated applications, reflecting adsorption-limited recoverability. However, it needs to be acknowledged that while matrix-matched calibration improves quantification accuracy, its implementation in routine monitoring may be challenging, particularly across diverse environmental conditions. Repeated soil application experiments further indicate that while naked dsRNA undergoes rapid degradation, chitosan-formulated dsRNA persists and accumulates to higher extractable levels, underscoring the necessity of explicitly considering formulation chemistry in environmental exposure assessments. Collectively, this study provides an adaptable RT-qPCR framework for environmental detection of dsRNA pesticides, highlights key methodological limitations, and provides a foundation for future studies to derive degradation kinetics (e.g., DT_50_ and DT_90_), assess additional formulations and matrices, and support standardized analytical strategies for regulatory risk assessment.

### Limitations of the study

This study examined a single dsRNA target and one chitosan-based formulation. Although the analytical framework is expected to be broadly applicable, further validation with additional dsRNA targets and formulations is needed. Due to the exploratory and method-development nature of the study, each treatment was represented by a single biological sample analyzed with multiple technical RT-qPCR replicates, reflecting the complexity of the extraction and calibration workflows. Assay specificity was supported by melting curve and sequencing analyses, although inclusion of unrelated non-target dsRNA controls in future studies would provide a more stringent specificity assessment. Finally, the study used a controlled worst-case application scenario; therefore, field studies under realistic agronomic conditions will be necessary to confirm environmental relevance.

## Resource availability

### Lead contact

Further information and requests should be directed to and will be fulfilled by the lead contact, Dr. Athanasios Dalakouras (nasosdal@gmail.com).

### Materials availability

This study did not generate new unique reagents.

### Data and code availability

• RT-qPCR data reported in this paper will be shared by the lead contact upon request.
• This paper does not report original code.
• Any additional information required to reanalyze the data reported in this paper is available from the lead contact upon request.

## Acknowledgments

This work was supported by the Horizon Europe project RATION (“Risk assessment of low-risk pesticides”) under grant number HORIZON-CL6-2022-FARM2FORK-01, HORIZON-RIA, no. 101084163. We sincerely thank the anonymous reviewers for their constructive comments and valuable suggestions, which significantly improved the quality and clarity of the manuscript.

## Author contributions

Conceptualization, K.K.P. and A.D.; methodology, V.K., D.G.K., K.K.P., and A.D.; investigation, V.K. and E.T.; writing – original draft, V.K.; writing – review and editing, V.K., M.K., D.G.K., K.K.P., and A.D.; funding acquisition, D.G.K., K.K.P., and A.D.; resources, M.K., D.G.K., K.K.P., and A.D.; supervision, K.K.P. and A.D.

## Declaration of interests

The authors declare no competing interests.

## STAR★Methods

### Key resources table

**Table:** 

REAGENT or RESOURCE	SOURCE	IDENTIFIER
**Bacterial and virus strains**		

Escherichia coli DH5α cells	New England Biolabs, NEB	Cat# C2987H

**Biological samples**		

Agricultural soil samples (“Ampelies”, “Kallipeuki”, “Larissa-University”)	This study	See coordinates in method details

**Chemicals, peptides, and recombinant proteins**		

DsRNA targeting *Leptinotarsa decemlineata actin* (dsACTIN)	Genolution Inc.	GenBank: KJ577616.1
Shrimp-shell chitosan (≥75% deacetylated)	Sigma-Aldrich	Cat# C3646
Sodium sulfate		
Sodium sulfate, ACS reagent, ≥99.0%, anhydrous, powder	Sigma-Aldrich	Cat# 238597
DNase I-XT	NEB	Cat# M0570S
RNase-If	NEB	Cat# M0243S
pGEM-T Easy Vector System	Promega	Cat# A1360
MOPS	Sigma-Aldrich	Cat# M1254
Sodium tetraborate decahydrate	Sigma-Aldrich	Cat# 31457
Sodium dihydrogen phosphate	Sigma-Aldrich	Cat# 1.06370
Phenol/Chloroform/Isoamyl Alcohol, 25:24:1 (v/v), Molecular Biology Grade	Sigma-Aldrich	Cat# 516726
Luna Universal One-Step Reaction Mix	NEB	Cat# E3005
Luna WarmStart RT Enzyme Mix	NEB	Included in Cat# E3005

**Critical commercial assays**		

RNeasy MinElute Cleanup Kit	Qiagen	Cat#74204
NucleoSpin Gel and PCR Clean-up Kit	Macherey-Nagel	Cat# 740609.50
NucleoSpin Plasmid Kit	Macherey-Nagel	Cat# 740588.50
RNeasy PowerClean Pro Cleanup Kit	Qiagen	Cat# 13997-50

**Oligonucleotides**		

Primers targeting dsACTIN, see method details	This study	N/A
M13 universal primer	Thermo Fischer Scientific	Cat# N52002

**Software and algorithms**		

Bio-Rad CFX Manager 3.1	Biorad	https://www.bio-rad.com/en-gr/category/thermal-cyclers-for-pcr?ID=75f1b406-3746-4580-a998-74245b094f56
GraphPad Prism 10.0.0 for Windows	Graphpad Software, Boston, MA, USA	https://www.graphpad.com/
Inkscape 1.3.1	91 b66b0; 2023-11-16	https://inkscape.org/
NCBI Primer-BLAST	NCBI	https://www.ncbi.nlm.nih.gov/tools/primer-blast/
OligoAnalyzer Tool	Integrated DNA Technologies, IDT	https://eu.idtdna.com/pages/tools/oligoanalyzer

**Other**		

Sanger sequencing data	This study	Available upon request

### Method details

#### DsRNA generation

The 297-bp dsRNA (dsACTIN) targeting *Leptinotarsa decemlineata actin* gene (GenBank: KJ577616.1) was synthesized by Genolution Inc., using T7-based *in vitro* transcription and purified via tangential flow filtration.^37^ dsACTIN was stored at −80°C in aliquots as a ready-to-use aqueous solution.

#### Chitosan formulation of dsRNA

Chitosan/dsACTIN nanoparticles (dsACTIN-chit) were prepared following established protocols (Sarathi et al., 2008; Ramesh Kumar et al., 2016). Essentially, shrimp-shell chitosan (Sigma-Aldrich, C3646, ≥75% deacetylated) was dissolved in 0.1 M sodium acetate buffer to yield a 0.02% (w/v) working solution. DsACTIN diluted in 0.05 M sodium sulfate buffer was then mixed with the chitosan solution to the desired mass ratio, heated to 55°C for 2 min, and vortexed for 60 s to facilitate nanoparticle formation.

#### Nuclease treatment of dsRNA

DsACTIN stock solution (8 × 10^9^ ng/L) was serially diluted to obtain a working concentration of 8 × 10^6^ ng/L. For nuclease treatment, 100 μL of the dsACTIN working solution (8 × 10^6^ ng/L) was used. Where applicable, chitosan was added at a 5:1 (w/w) ratio prior to enzymatic treatment. To remove contaminating DNA, dsACTIN was treated with DNase I-XT (New England Biolabs, M0570S) for 45 min at 37°C, followed by purification using the RNeasy MinElute Cleanup Kit (Qiagen, 74204). Subsequently, ssRNA was removed via RNase-If treatment (NEB, M0243S) at 37°C for 20 min, followed by a second cleanup step.

#### RT-qPCR detection

Primers targeting a 121 bp dsACTIN fragment were designed using NCBI Primer-BLAST and OligoAnalyzer (IDT): forward 5′-GAT GTA GCG GCT CTT GTC GT-3′, reverse 5′-TGA CTC CTT GAT GCC TTG GG-3’. Denaturation and primer annealing were performed by heating 38 μL sample with 5 μL of each primer (10 μM) at 95°C for 5 min, followed by −20°C for 15 min. The 10 μL RT-qPCR reaction contained 4.5 μL sample-primer mix, 5 μL 2× Luna Universal One-Step Reaction Mix, and 0.5 μL Luna WarmStart RT Enzyme Mix (NEB, E3005). Reactions were conducted on a CFX Connect Real-Time PCR Detection System (Bio-Rad) with the following thermal profile: 55°C for 10 min (RT), 95°C for 60 s (initial denaturation), 40 cycles of 95°C for 30 s and 62°C for 30 s. Melting curve analysis (55°C–95°C, 0.05°C–0.5°C increments) confirmed product specificity. Ct values were determined using Bio-Rad CFX Manager 3.1. Due to the fundamental design of the study as a methodological assessment, for each experimental condition, one biological sample was processed and analyzed by RT-qPCR in three technical replicates. Negative controls included dsRNA buffer-only samples (naked dsRNA control) and dsRNA buffer mixed with chitosan buffer in the absence of dsRNA (formulated dsRNA control). Linear regression analysis of standard curves was performed using GraphPad Prism version 10.0.0 for Windows (GraphPad Software, Boston, MA, USA), and 95% confidence intervals for slope parameters were calculated for each curve.

#### Sanger sequencing of RT-qPCR amplicons

RT-qPCR products (121 bp) were separated on 1% agarose TAE gel, excised, and purified using the NucleoSpin Gel and PCR Clean-up Kit (Macherey Nagel, 740609.50). Amplicons were cloned into pGEM-T Easy vector (Promega, A1360), transformed into *E. coli* DH5α, and five plasmids from individual colonies were extracted (NucleoSpin Plasmid Kit, Macherey-Nagel, 740588.50) for Sanger sequencing using the M13 universal primer (Thermo Fischer Scientific, Cat# N52002).

#### DsRNA application and extraction from soil

DsACTIN and dsACTIN-chit were spiked onto 0.3 g soil at concentrations ranging from 10^−3^ to 10^4^ ng_dsRNA_/g_soil_ (ng g^−1^) using a 1.5:1 chitosan:dsRNA mass ratio. In the study there were included (i) topsoil from Kallipeuki, Thessaly, Greece (pH 6.7; sandy loam; 14% clay, 32% silt, 54% sand; organic C content 2.7%, *N* 39.962335, E 22.461202), (ii) topsoil from Ampelies, North Greece (pH 4.0; sandy loam; 11% clay, 27% silt, 62% sand; Organic C content 1.03%, *N* 40.850340, E 22.375556), and (iii) topsoil from the campus of the University of Thessaly, Larissa, Greece (pH 8.3; sandy loam; 28.4% clay, 20% silt, 51.6% sand; OC 0.62, *N* 39.613972, E 22.389472; pH 8.3). Samples were incubated in 0.6 mL of MOPS-extraction buffer (3 mM MOPS, 10 mM NaCl, pH adjusted to native soil) for 2 h at 24°C on an overhead rotator. Following centrifugation (21,100 × g, 5 min), the supernatant (named ‘soil solution’) was separated. Soil pellets were then treated with 0.875 mL orthophosphate-tetraborate buffer (12 mM orthophosphate, 200 mM tetraborate, 10 mM NaCl, pH 11) at 4°C for 4 h to recover particle-associated dsRNA (named ‘soil extract’). Both fractions were neutralized and subjected to phenol:chloroform:isoamyl alcohol (25:24:1, v/v) purification, followed by isopropanol precipitation at −20°C for 16 h. Pellets were washed twice with 0.5 mL cold ethanol, dried, and resuspended in 0.1 mL buffered solution (3 mM borate, 10 mM NaCl, 12 mM phosphate, pH 7.0). Column purification was performed using the RNeasy PowerClean Pro Cleanup Kit (Qiagen, 13997–50). Soil solutions and extracts were diluted 100× and 10×, respectively, prior to RT-qPCR analysis. Different dilution factors were initially tested to evaluate their impact on RT-qPCR inhibition and detection sensitivity. The selected dilution ratios represented a compromise between reducing matrix-associated inhibition and preserving sufficient dsRNA signal for reliable quantification. For each experimental condition, one biological soil sample was processed and analyzed by RT-qPCR in three technical replicates.

### Quantification and statistical analysis

RT-qPCR quantification was performed using standard curves generated from serial dilutions of dsACTIN in both *in vitro* preparations and soil matrix-derived samples. Ct values were obtained using Bio-Rad CFX Manager 3.1, while linear regression analyses were conducted using GraphPad Prism version 10.0.0 (GraphPad Software, Boston, MA, USA). Regression parameters, including regression equations, slopes, coefficients of determination (R^2^), amplification efficiencies, and corresponding 95% confidence intervals (CI) of the slopes, are provided in Table S1 and indicated in the relevant figure legends. For each experimental condition, one biological sample was processed and analyzed by RT-qPCR in three technical replicates. Only Ct values within the defined dynamic range and showing consistent amplification behavior were included in regression analyses, whereas non-detectable or inconsistent values were excluded. Product specificity was evaluated by melting curve analysis and Sanger sequencing of representative RT-qPCR amplicons. Since the study focused on methodological optimization rather than hypothesis-driven comparisons, data were analyzed descriptively without formal inferential statistical testing between treatments.
