# Supplementary material for: RT-qPCR detection of dsRNA pesticides: Optimization, limitations, and formulation effects
Source: iScience. 2026 Jun 18;29(7):116428. doi: 10.1016/j.isci.2026.116428 (PMC13312021; doi:10.1016/j.isci.2026.116428)
Supplement: Document S1. Figures S1–S3 and Tables S1–S4 [file mmc1.pdf]

iScience, Volume *29*

Supplemental information

RT-qPCR detection of dsRNA pesticides: Optimization, limitations, and formulation effects

Venetia Koidou, Evangelia Tamvakologou, Minlee Kim, Dimitrios G. Karpouzas, Kalliope K. Papadopoulou, and Athanasios Dalakouras

SUPPLEMENTAL INFORMATION

Document S1. Figures S1-3, Tables S1-4

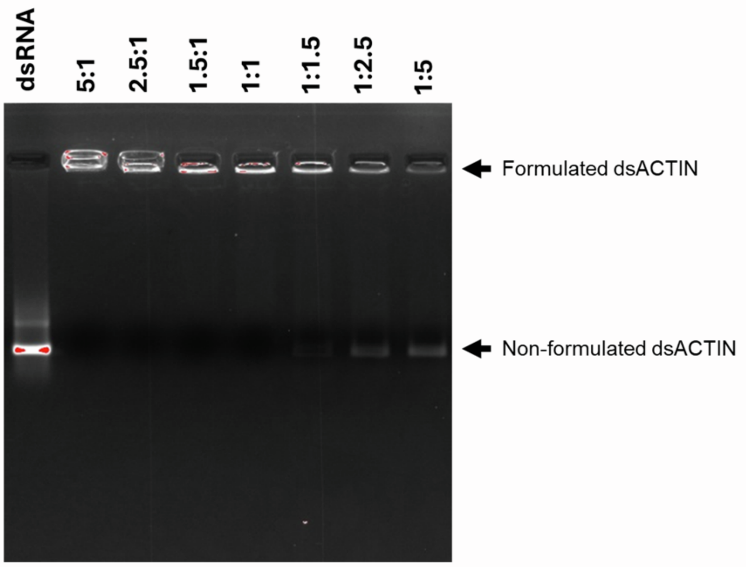

Figure S1. Gel retardation assay of chitosan-dsACTIN complexes at different mass ratios. Approximately 250 ng of dsACTIN was formulated with chitosan at various chitosan:dsACTIN mass ratios (5:1 to 1:5) and analyzed by electrophoresis on a 1% agarose TAE gel. Non-formulated dsACTIN was loaded in the first lane as a control. Free dsRNA migrated according to its molecular size, whereas chitosan-bound dsRNA exhibited reduced electrophoretic mobility or complete retention in the gel wells.

```
CLUSTAL O(1.2.4) multiple sequence alignment


qPCR-amplicon      ------------------------------------------------------------ 0
dsACTIN            GCACGAGGTTTTTCTGTCTAGTGAGCAGTGTCCAACCTCAAAAGACAACATGTGTGACGA 60


qPCR-amplicon      -GATGTAGCGGCTCTTGTCGTAGACAATGGATCCGGTATGTGCAAAGCCGGTTTCGCAGG 59
dsACTIN            CGATGTAGCGGCTCTTGTCGTAGACAATGGATCCGGTATGTGCAAAGCCGGTTTCGCAGG 120
                    ***********************************************************

qPCR-amplicon      AGATGACGCACCCCGTGCCGTCTTCCCCTCGATCGTCGGTCGCCCAAGGCATCAAGGAGT 119
dsACTIN            AGATGACGCACCCCGTGCCGTCTTCCCCTCGATCGTCGGTCGCCCAAGGCATCAAGGAGT 180
                   ************************************************************

qPCR-amplicon      CA---------------------------------------------------------- 121
dsACTIN            CATGGTCGGTATGGGACAAAAGGACTCATACGTAGGAGATGAAGCCCAAAGCAAAAGAGG 240
                   **

qPCR-amplicon      ---------------------------------------------------------   121
dsACTIN            TATCCTCACCCTGAAATACCCCATCGAACACGGTATCATCACCAACTGGGATGACAT   297
```

Figure S2. **Sanger sequencing validation of RT-qPCR amplicons.** RT-qPCR amplicons were cloned to plasmid vectors and subjected to Sanger sequencing. Sequence analysis (here one of them is presented as an example) confirmed that the amplified products corresponded to the expected dsACTIN fragment (GenBank accession no. KJ577616.1). Sequence alignment was performed using CLUSTAL Omega.

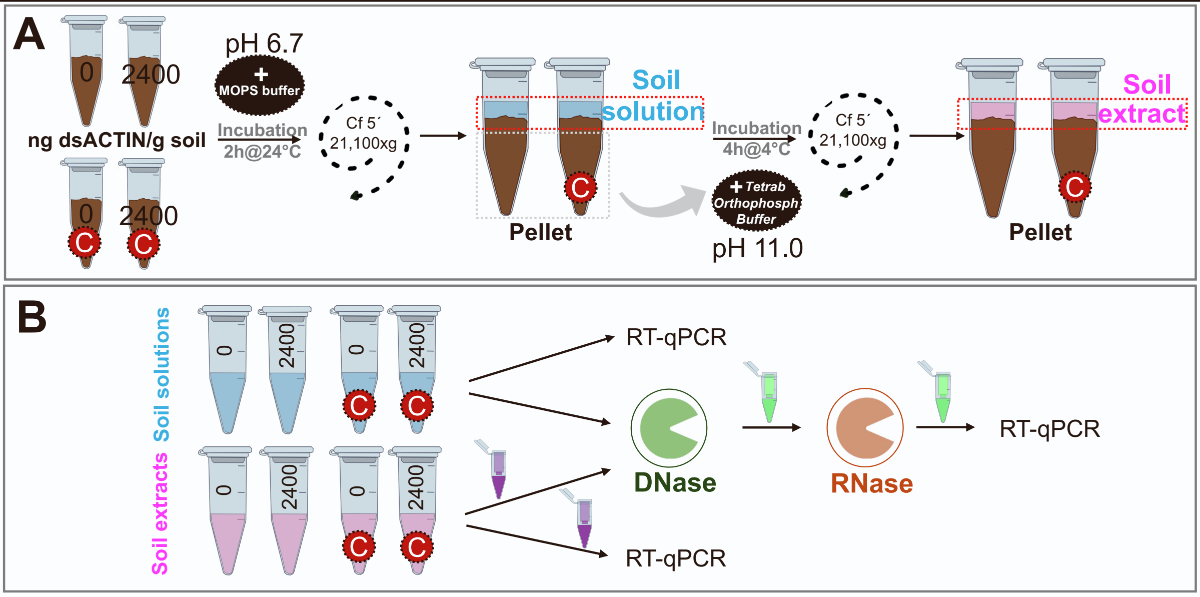

C

DOSE (ng g^{-1} dry soil)	Soil Extracts			
	**dsACTIN** (ng g^{-1} dry soil)			
**CHITOSAN**	-		+	
**NUCLEASES**	-	+	-	+
**0**	0,004	0,212	0,069	2,285
**2400**	9232,811	939,575	152917,894	1227,923

D

DOSE (ng g^{-1} dry soil)	Soil Solutions			
	**dsACTIN** (ng g^{-1} dry soil)			
**CHITOSAN**	-		+	
**NUCLEASES**	-	+	-	+
**0**	0,006	0,007	0,031	0,437
**2400**	0,021	0,245	0,130	0,718

Figure S3. RT-qPCR detection of dsACTIN from soil samples and assessment of nuclease treatment and formulation effects using *in vitro* standard curves. A.) Experimental workflow for dsRNA application and recovery from soil. Naked and chitosan-formulated dsACTIN (chitosan:dsACTIN 1.5:1) was applied to soil at 2400 ng g^{-1} dry soil. Following incubation, dissolved dsRNA was recovered as the 'soil solution' fraction, while adsorbed dsRNA was desorbed using alkaline orthophosphate buffer and recovered as the 'soil extract' fraction. B.) Schematic of downstream processing. Both fractions were subjected to sequential DNase I-XT and RNase-If treatments, followed by column purification and RT-qPCR analysis. C.) Quantification of dsACTIN recovered from soil extract fractions under the indicated conditions (presence or absence of nucleases and/or chitosan formulation). D.) Quantification of dsACTIN recovered from soil solution fractions under the indicated conditions (presence or absence of nucleases and/or chitosan formulation). Values are expressed as ng dsACTIN g^{-1} dry soil. For each condition, the concentrations were estimated using in vitro standard curves. Panels A and B were prepared in Inkscape 1.3.1 (91b66b0, 2023-11-16). -C corresponds to naked dsRNA, +C corresponds to dsRNA formulated with chitosan.

DOSE (ng/L)	dsACTIN	dsACTIN-chit	dsACTIN-nuc	dsACTIN-nuc/chit
8 x 10^6	ND	ND	**7,11**	**17,94**
8 x 10^4	**7,79**	**7,54**	**13,26**	**22,39**
8 x 10^2	**11,59**	**11,86**	**17,40**	**23,89**
8	**14,02**	**14,77**	**17,58**	**24,94**
8 x 10^{-1}	**16,95**	**20,43**	**21,90**	**25,74**
8 x 10^{-2}	**21,78**	**22,18**	26,60	30,19
8 x 10^{-3}	22,91	**22,25**	27,39	30,23
8 x 10^{-4}	26,42	24,27	28,64	30,51
8 x 10^{-6}	26,52	ND	30,17	30,55
8 x 10^{-8}	29,04	ND	30,27	ND

Table S1. ***In vitro* standard curves for dsACTIN under different treatment conditions.** Serial dilutions of dsACTIN subjected to different treatments (dsACTIN, dsACTIN-nuc, dsACTIN-chit, and dsACTIN-nuc/chit) were analyzed by RT-qPCR to evaluate detection linearity and sensitivity. Ct values were plotted against dsRNA concentrations ranging from 8 × 10^6 to 8 × 10^{-8} ng/L. Linear regression equations and coefficients of determination (R^2) were calculated for each standard curve and regression parameters, including slope and corresponding 95% confidence intervals (CI), are provided in **Table S4**. Bold values indicate the data points included in the regression analyses. The standard curves illustrate the quantitative response of each treatment and allow comparison of trends associated with nuclease treatment and chitosan formulation. ND indicates values below the limit of detection (non-detectable). Negative controls (formulation and buffer-only controls without dsRNA) showing occasional non-specific amplification were excluded from regression analysis.

	Acidic soil				Neutral Soil				Alkaline Soil			
**FRACTION**	**S**		**E**		**S**		**E**		**S**		**E**	
**CHITOSAN**	**-**	**+**	**-**	**+**	**-**	**+**	**-**	**+**	**-**	**+**	**-**	**+**
**NOMINAL DOSE dsACTIN** (ng g⁻¹ dry soil)												
0	29,04	28,96	ND	37,53	24,56	24,31	24,01	24,14	27,16	28,81	26,33	26,21
0	30,08	29,63	38,05	36,05	24,27	24,38	24,27	24,4	27,33	28,61	26,29	26,25
10^{-3}	**30,42**	**30,11**	**23,47**	**31,79**	**24,72**	**24,56**	**24,63**	**24,34**	**27,85**	**28,95**	**26,35**	**26,18**
10^{-2}	**30,05**	**29,84**	**21,38**	**30,48**	**24,51**	**24,38**	**23,43**	**23,73**	**27,39**	**28,91**	**25,68**	**22,66**
10^{-1}	**30,08**	**29,38**	**17,35**	29,93	**24,21**	**24,29**	**20,79**	**24,06**	**27,38**	**28,87**	**21,64**	**20,9**
10^0	29,97	**28,74**	17,66	**21,58**	**24,18**	**24,23**	**20,48**	**19,8**	**27,31**	**28,79**	**20,52**	**20,39**
10^{1}	29,89	**28,65**	**12,53**	20,68	**24,14**	**24,21**	**16,3**	**17,92**	**27,25**	28,75	**19,27**	**13,38**
5×10^{1}	29,52	**28,33**	**12,67**	20,56	**24,08**	**24,19**	**16,39**	**16,34**	27,21	28,68	**18,77**	**9,81**
10^{2}	**27,67**	**28,16**	**12,47**	7,78	**24,06**	**24,17**	**11,35**	**12,37**	27,13	**26,2**	**13,17**	**7,07**
5×10^{2}	**25,93**	**28,03**	11,33	7,22	22,78	24,15	**8,96**	**7,43**	26,57	**25,26**	**8,75**	**5,7**
10^{3}	**24,65**	**27,59**	**7,43**	**7,88**	22,03	24	2,0	5,75	**22,95**	**23,54**	**4,53**	**3,8**
10^{4}	**23,93**	**26,77**	**3,87**	**3,69**	21,99	24,03	ND	3,64	**22,5**	**22,78**	**1,27**	**1,1**

Table S2. Matrix standard curves for dsACTIN spiked into different agricultural soils. Matrix standard curves were generated by spiking naked (−C) and chitosan-formulated (+C) dsACTIN and dsACTIN-chit, respectively, into soil samples at concentrations ranging from 10^{-3} to 10^{4} ng g^{-1} dry soil and processing them through the complete soil extraction workflow. Ct values obtained by RT-qPCR from soil solution (S) and soil extract (E) fractions were plotted against log10-transformed dsRNA concentrations. Linear regression analyses were used to construct matrix standard curves, enabling accurate quantification of dsRNA recovered from experimental soil samples. Matrix-matched negative controls, containing all buffer and formulation components but no dsRNA, were included to account for background amplification. Ct values were included in regression analysis only when falling within the defined dynamic range and exhibiting consistent amplification behaviour. The resulting regression parameters, including regression equations, slopes and corresponding 95% confidence intervals (CI), are provided in **Table S4**. Bold values indicate data points included in the regression analyses. ND denotes values below the limit of detection (non-detectable).

**Dose** (ng g^{-1} dry soil)	**0**		**60**	
**CHITOSAN**	**-**	**+**	**-**	**+**
**TIME**				
**t0**	7,66E-04	4,51E-03	1,39E-01	1,04E-02
	**1,75E-34**	**1,22E-59**	**1,70E-01**	**2,92E-47**
**t7**	8,23E-04	6,62E-03	1,09E-02	1,97E-02
	**6,60E-34**	**6,14E-54**	**2,99E-13**	**9,29E-38**
**t14**	1,56E-03	1,28E-02	3,21E-02	3,59E-02
	**8,29E-29**	**4,29E-44**	**1,32E-04**	**7,69E-29**
**t21**	9,57E-04	5,64E-03	1,22E-01	2,57E-03
	**1,07E-32**	**2,52E-56**	**1,56E-02**	**5,07E-68**
**t28**	5,42E-04	7,80E-03	8,39E-02	1,65E-02
	**3,00E-37**	**1,68E-51**	**1,47E-05**	**2,17E-40**
**t42**	1,29E-03	4,35E-02	5,56E-02	2,48E-02
	**2,63E-30**	**5,77E-26**	**1,05E-09**	**2,39E-34**

Table S3. **RT-qPCR detection of dsRNA in soil following repeated applications of naked and chitosan-formulated dsRNA using a minimized workflow: soil solution fractions.** Quantification of dsACTIN recovered from soil solution fractions after repeated applications of naked and formulated dsRNA. Results are expressed as ng g^{-1} dry soil. For each treatment, values in the upper row represent concentrations calculated using *in vitro* standard curves, whereas values in the lower row (bold) represent concentrations calculated using matrix-derived standard curves.

Treatment	Matrix	Equation	Slope	95% CI (slope)	R^2
**dsACTIN**	*In vitro*	Y = -2,131*X + 17,63	-2,131	-3,162 to -1,100	0,9352
**dsACTIN-nuc**	*In vitro*	Y = -1,870*X + 21,25	-1,87	-2,861 to -0,8794	0,9232
**dsACTIN-chit**	*In vitro*	Y = -2,340*X + 18,54	-2,34	-3,700 to -0,9798	0,909
**dsACTIN-nuc/chit**	*In vitro*	Y = -1,023*X + 26,15	-1,023	-1,643 to -0,4021	0,9017
**dsACTIN (-C)**	Soil solution (Ampelies-acidic)	Y = -0,9498*X + 28,31	-0,9498	-1,284 to -0,6155	0,9143
**dsACTIN (+C)**	Soil solution (Ampelies-acidic)	Y = -0,4378*X + 28,93	-0,4378	-0,5090 to -0,3666	0,9617
**dsACTIN (-C)**	Soil extract (Ampelies-acidic)	Y = -2,637*X + 15,78	-2,637	-3,107 to -2,168	0,9692
**dsACTIN (+C)**	Soil extract (Ampelies-acidic)	Y = -4,169*X + 20,88	-4,169	-4,690 to -3,648	0,992
**dsACTIN (-C)**	Soil solution (Kallipeuki-neutral)	Y = -0,1157*X + 24,26	-0,1157	-0,1731 to -0,0582	0,8428
**dsACTIN (+C)**	Soil solution (Kallipeuki-neutral)	Y = -0,06836*X + 24,28	-0,0684	-0,0978 to -0,0389	0,8768
**dsACTIN (-C)**	Soil extract (Kallipeuki-neutral)	Y = -2,606*X + 18,25	-2,606	-3,477 to -1,734	0,8992
**dsACTIN (+C)**	Soil extract (Kallipeuki-neutral)	Y = -2,751*X + 18,73	-2,751	-3,922 to -1,580	0,8463
**dsACTIN (-C)**	Soil solution (Larissa-alkaline)	Y = -0,8063*X + 26,32	-0,8063	-1,270 to -0,3430	0,8001
**dsACTIN (+C)**	Soil solution (Larissa-alkaline)	Y = -0,9339*X + 27,33	-0,9339	-1,305 to -0,5632	0,8636
**dsACTIN (-C)**	Soil extract (Larissa-alkaline)	Y = -3,535*X + 18,96	-3,535	-4,672 to -2,399	0,8654
**dsACTIN (+C)**	Soil extract (Larissa-alkaline)	Y = -3,807*X + 16,30	-3,807	-4,412 to -3,202	0,9634

Table S4. Linear regression parameters of in vitro and matrix-derived standard curves used for RT-qPCR quantification of dsRNA. Linear regression analysis was performed using GraphPad Prism. Reported parameters include regression equation, slope, coefficient of determination (R^2), and corresponding 95% confidence intervals (CI) of the slope.
